# Matrices

**Published:** 1888-02

**Authors:** 


### Editorial.

### MATRICES.

In the *Dental Cosmos* for April, 1871, Dr. Louis Jack, of Philadelphia, gave a description and illustrative cuts of what was to us a new appliance to be used in the filling of teeth. It was called a dental matrix. Statistics, hastily compiled from the remarks made at dental society meetings, have since compelled the belief that about 4,000 dentists had preceded Dr. Jack in the use of that device, and had "been using it for years." It was eminently one of those inventions which the 4,000, more or less, "had not thought it worth while to mention to any one," but which, when it was mentioned, wrought a revolution in the practice of many dentists. It has proved of sufficient importance to employ the inventive talent of some of the best men in dentistry, in an effort to improve it and to extend its capabilities. We commenced its use very soon after its introduction, and have continued to employ it to this day.

Practically, the matrix may be defined as a temporary wall added to a compound cavity, thus reducing it to a simple one. Its use appears exceedingly easy, but in actual practice it will be found that it will absorb all the skill and dexterity possessed by our best operators. Dr. G. C. Daboll, formerly of Buffalo, now of Paris, France, than whom America has produced few more accomplished operators, and who commenced with it more than fifteen years ago, was accustomed to say that while to use it was the most elementary of processes, to use it well, and to obtain from it all the advantages which it was capable of conferring, taxed his ingenuity as much as that of any appliance in his operating case. This fact undoubtedly accounts for the failure of many skillful dentists who have attempted to employ it. They could succeed in inserting a filling by its aid, but they felt that the work was not equal to that which they could do without it. Could we personally have foreseen from the start the number of failures which, in our own practice, would have been the result of its use, it is doubtful if we should ever have taken it up. And yet, to-day, it is an essential, and there is a class of cavities which we should hardly know how satisfactorily to fill without it.

We have heard objections urged against the matrix which clearly proved that the objectors knew not its limitations, or the cases in which it was especially useful, for it is not every approximal filling which demands its employment. The beginner, indeed, will do well to restrict it to a very limited number of cases, advancing to those more difficult as he gains in experience. It is a mistake to attempt to apply the matrix in perplexing operations until experience has been gained, for ultimate failure will be the sure result. One of the greatest obstacles to success in its employment is the great difficulty in adapting it to teeth having irregular forms and surfaces, and to those from which a part of the wall against which it should impinge is broken away. In adjusting it the rubber-dam must of course be first applied, and then if an unyielding matrix is used, it must be wedged so that it will be absolutely immovable. To secure this we use only thin matrices, and wedge them fast by slips of orange wood dipped in a sandarach solution, wedging from both the lingual and labial surfaces.

The preparation of the cavity for which a matrix is to be used requires great judgment. It is absolutely essential that every part of the cavity should be exposed to the direct action of the plugger. If there be deep undercuts, especially under the wall next the operator, the gold will not be solidly condensed beneath, and failure of the operation will be certain. The cavity must be opened upon the grinding surface to its full size, and if it be large the anchorage should be mainly by dovetails in the crown. When, for any reason, it would be bad practice to cut away the grinding surface of a tooth to this extent, the matrix should not be employed.

It is exceedingly difficult in many cases to secure the lateral marginal walls where the matrix is used. With the old inflexible steel matrix, this was sometimes impossible. If the matrix was firmly wedged against the tooth the gold could not be carried over the frangible wall, and thus be made to act as a support, and for these cases the inflexible matrix was not adapted. We have heard it urged against the matrix that contour could not be secured, but that the filling would be left flat. If this was found to be the case, the fault certainly was with the operator and not with the implement, for, given a tooth with firm lateral walls, the most beautiful and natural shapes and forms can be secured with little difficulty and no waste of material, while the solidity of the whole filling, its close adaptation to the cervical wall and the density of its masticating surface, may be made such as can scarcely be attained by any other means.

Within a very few years new forms of matrices have been devised, that are for many cases great improvements over the original patterns. Bands, with various devices for drawing them closely about the tooth, seem to be the favorites. The Lardmore-Brunton clamp-matrix is one of the best of these. It is an English device, and may be obtained of C. Ash & Sons, 30 East 14th Street, New York. Guilford's band matrix presents some advantages. It is made by the S. S. White Dental Manufacturing Company. Each of these consists of a band which is placed about the tooth, the ends being drawn together by a screw clamp. Dr. Herbst makes a matrix for each case by drawing about the tooth a German silver band, pinching it together by nippers devised for the purpose, and then soldering it. But of all the band matrices with which we have had any experience, that of Dr. T. W. Brophy, of Chicago, is the best. It is simply a flexible belt to be placed about the tooth, one side of which is made of sufficient thickness to afford a thread for a screw which is driven against the tooth, thus drawing the matrix tight and causing it to conform to almost any irregular contour. It is made in six sizes, so that any tooth can be embraced by it.

Some of the advantages of Brophy's matrix are, its perfect ease of adaptation and the exactness of its fit at all essential points when it is perfectly adjusted. If a portion of one of the lateral walls is gone, the Brophy matrix makes its restoration easy and simple. If it be the lingual wall that is broken down, the matrix should be adjusted with the screw upon the labial side; while, if the labial surface be missing, the matrix should be reversed and the screw be adjusted to the lingual wall. To turn the screw in such cases a key with a flexible joint, like that of the Lardmore-Brunton clamp, is essential. Contour is easily secured, missing walls restored and the gold built over frail walls, if the matrix be perfectly adjusted. In cases of restoration, we are accustomed, when we approach the "knuckle," to slightly loosen the screw and thus give more space. But so flexible is the band that if the gold be driven against one portion a little more forcibly than against the rest, the contour is easily and readily swelled.

But let no one who is without long experience take up the matrix and expect to obtain perfect results at the first effort. It requires great familiarity with its method of manipulation, and its perfect adaptation is no more easily acquired than is that of the rubber-dam, the Perry separator, or the manipulation of the electric mallet.

